# A Systematic Review of Online Medical Consultation Research

**DOI:** 10.3390/healthcare12171687

**Published:** 2024-08-23

**Authors:** Tian Shen, Yu Li, Xi Chen

**Affiliations:** 1 School of International Education, Nanjing University of Chinese Medicine, Nanjing 210023, China; shentian025@njucm.edu.cn; 2 Business School, Nanjing University, Nanjing 210093, China; doctor_chan@163.com

**Keywords:** online medical consultation, online medical platform, doctors, patients, systematic review

## Abstract

Online medical consultation is a form of medical service that facilitates interactions between patients and doctors online, offering significant utility and value. This review aims to retrieve, screen, and analyze articles related to online medical consultations, formulating a theoretical framework and proposing future research directions. According to PRISMA guidelines, a systematic search was conducted in Web of Science, EBSCO, ScienceDirect, PubMed, and Scopus, retrieving a total of 4072 English records on 16 December 2023. After rigorous screening, 75 articles were included in this review. Among these, 8 articles focused on patients utilizing online medical consultation platforms, 5 on doctors participating in online medical platforms, 18 on patients’ choice of doctors, 12 on doctors providing services, 7 on online reviews of patients, 14 on service quality for patients, 8 on rewards to doctors, and 11 on the spillover effect between online and offline services. These themes comprise the theoretical framework of the starting point, process, and outcomes of the online medical consultation system, providing a comprehensive understanding of the field and a foundation for future research.

## 1. Introduction

In the era of digitalization and COVID-19, the landscape of healthcare delivery is undergoing a significant transformation with the flourishing of digital healthcare. This transformation is primarily evident in the escalating trend of patients seeking healthcare-related advice and consultations through digital platforms, shifting from traditional offline outpatient services to online medical consultations [1]. The digitalization of healthcare has brought about a significant transformation in the delivery of healthcare services, particularly in the shift from traditional offline outpatient services to online solutions for addressing patients’ health issues [2]. This paradigm shift holds the potential for enhancing the cost-effectiveness of healthcare service delivery while also improving the overall user experience [3]. Consequently, the approach of online medical consultation has gained widespread popularity on a global scale. The projected revenue for the online medical consultations segment is estimated to reach USD 25.31 billion in 2023, with an anticipated annual growth rate of 8.76%. This growth trajectory is expected to contribute to a market volume of approximately USD 35.41 billion by 2027 [4]. Online medical consultation has emerged as a novel service channel in the healthcare domain, effectively bridging the information gap between doctors and patients. This innovative approach complements traditional healthcare services, alleviates the burden on the healthcare system, and facilitates the equitable allocation of healthcare resources, which draws significant attention from both academic researchers and industry practitioners [5].

**Figure 1:**
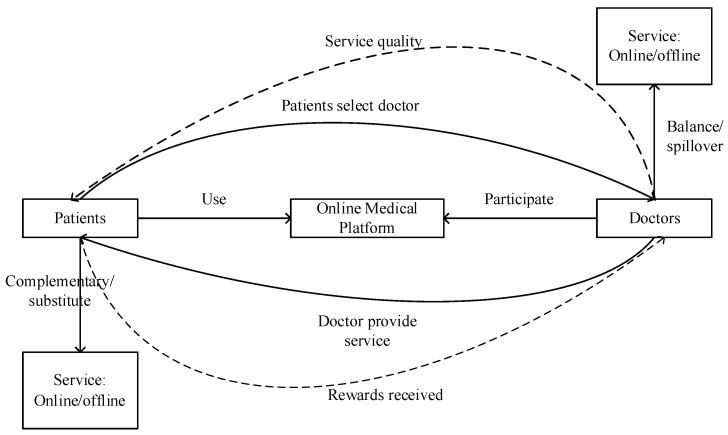
Online medical consultation system.

Online medical consultation can be defined as a novel medical service model that enables doctors and patients to engage in health-related discussions anytime and anywhere through online medical platforms, utilizing various forms of communication such as text, pictures, voice, and video, facilitated by electronic devices [6,7,8,9,10]. The definition encompasses three core elements that constitute the online medical consultation system (illustrated in Figure 1). Firstly, the technical systems: online medical platforms. Online medical websites and platforms developed in the healthcare field are the carriers of Internet healthcare innovation, online medical consultation is one of the online health services provided by websites [11]. Secondly, the social systems: both doctors and patients as key participants. In online medical consultation, healthcare professionals, including doctors, and individuals such as patients or their families, play crucial roles, with doctors serving as service providers and patients as service recipients [6]. Thirdly, results of the integration of social and technical systems: doctor–patient interaction processes. The main methods of doctor–patient interaction include text, pictures, voice, and video. During these interactions, patients actively provide information about their health conditions and pose inquiries [7], while doctors offer professional medical guidance to address concerns [12]. Following the completion of the service, patients may also provide feedback on the quality of the doctor’s service, and doctors get rewarded for their services. Therefore, online medical consultations are a sociotechnical system that has the characteristic of cross-regional [8], timelines [6], convenience [12], professionalism [9], and high value [13].

Research related to online medical consultation has been increasing in recent years and has presented the importance of online medical consultation in the healthcare system. However, the existing literature lacks cohesion and organization, with a dearth of systematic sorting and integration of studies pertaining to online medical consultation. While Lu et al. (2022) conducted a bibliometric review on research related to online medical consultation, Wang et al. (2022) investigated the antecedents and consequences of online healthcare community usage using grounded theory [5,14]. No comprehensive overview or summary of the specific elements focused on online medical consultation research has been conducted thus far. Building upon the existing research findings in this domain, this paper aims to systematically review the current state of research and develop future perspectives on online medical consultation. By doing so, it intends to establish a more comprehensive and cohesive framework, serving as a reference for future research endeavors in the field of online medical consultation.

## 2. Materials and Methods

To comprehensively examine the existing literature on online medical consultations, a systematic review following the Preferred Reporting Items for Systematic Reviews and Meta-Analyses (PRISMA) guidelines is considered a suitable approach for investigating the current state of research in this domain [15].

To search existing research, we retrieve the literature by relevant terms in the following five major databases: Web of Science, EBSCO, ScienceDirect, PubMed, and Scopus. These terms include: “online health/healthcare/medical community (ies)”, “online health/healthcare/medical consultation (s)”, “online health/healthcare/medical counseling”, “online health/healthcare/medical service (s)”, and “online health/healthcare/medical platform (s)”. The search was conducted on 16 December 2023. Furthermore, to ensure a comprehensive identification and screening of potentially relevant articles, a snowballing technique was employed. This iterative method involves using the reference lists of already retrieved articles as a starting point to locate additional relevant studies. By pursuing this approach, we aimed to minimize the risk of overlooking important literature in the field of interest.

The eligibility criteria for this systematic review are published articles and reviews. Then, we apply the following selection criteria: (1) the articles were written in English; (2) the journal to which the article belongs is either a leading journal in the field or has a higher impact factor; (3) the articles employed qualitative or quantitative research methods; (4) the articles addressed topics pertaining to online medical consultations; and (5) the articles focused on online healthcare communities involving both doctors and patients as active participants. This rigorous selection process aimed to ensure the inclusion of relevant and high-quality studies in our review.

## 3. Results

**Figure 2:**
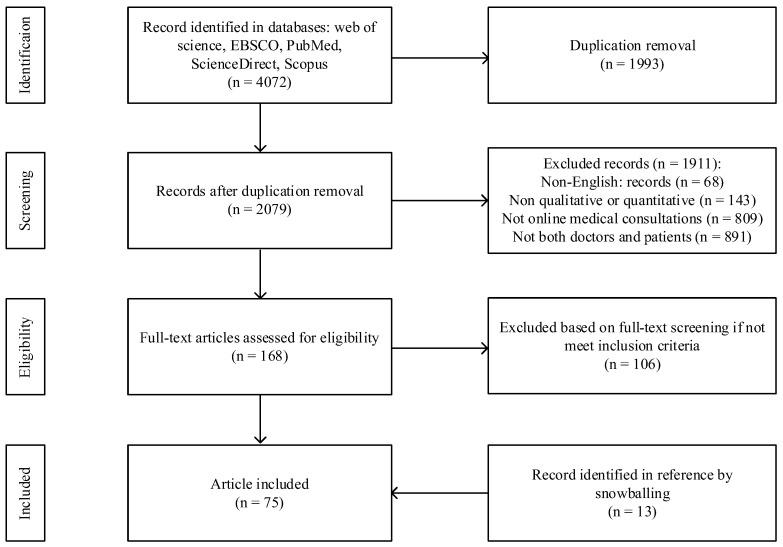
Flowchart of the literature search.

According to the PRISMA guidelines [16], we conducted a search and screening of articles related to online medical consultation. The initial literature search retrieved a total of 4072 papers, which were subsequently reduced to 2079 after eliminating duplicate records. Subsequently, two authors independently reviewed the titles and abstracts of the remaining articles following the selection criteria. If any disagreements arose, the third author would make the final judgment. During this process, 68 non-English records were excluded, 143 records were excluded for not employing qualitative or quantitative research methods, 809 articles were excluded because their topics were not related to online medical consultations, and 891 articles were excluded because they did not study online medical communities involving both doctors and patients. Therefore, 168 articles remained. These articles were then thoroughly examined to evaluate their research objectives, research methods, and main findings. As a result, 62 articles met the inclusion criteria based on their quality and relevance. Additionally, 13 eligible articles were identified through reference checking and snowballing, bringing the final number of included articles to 75. The specific process is shown in Figure 2.

The authors conducted a comprehensive review of the research objectives and content of the articles included in the systematic review, categorizing them into the following themes: (1) patients utilizing online medical platforms; (2) doctors participating in online medical platforms; (3) patient choice of doctor; (4) doctors providing services; (5) online reviews of patients; (6) service quality for patient; (7) rewards to doctors; (8) spillover effect between online and offline services. The aforementioned themes constitute the starting point, process, and outcomes of online medical consultations, thereby forming a comprehensive theoretical framework of the sociotechnical system, with the technical system as the foundation and the social system as the core.

### 3.1. Year of the Included Papers

**Figure 3:**
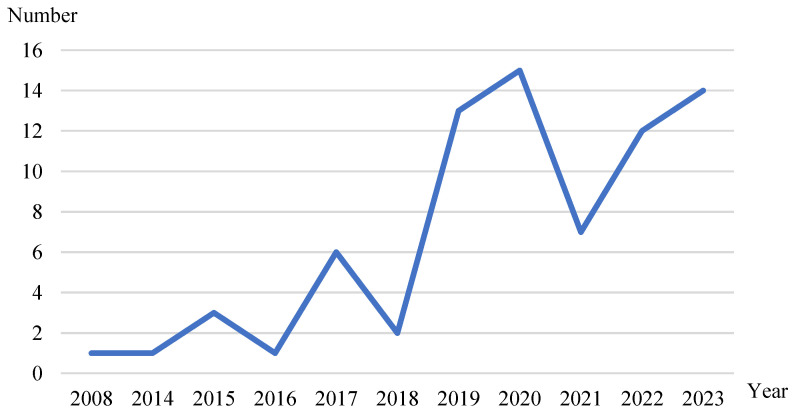
Distribution of the number of articles.

The papers included in the review were published between 2008 and 2023, as shown in Figure 3. Papers published from 2008 to 2016 accounted for 8%, those from 2017 and 2018 accounted for 10.67%, those from 2019 accounted for 17.33%, those from 2020 accounted for 20%, those from 2021 accounted for 9.33%, those from 2022 accounted for 16%, and those from 2023 accounted for 18.67%. This indicates that the field of online medical consultations has experienced rapid growth since 2019, becoming a focal point of research interest.

### 3.2. Methods of the Included Papers

**Figure 4:**
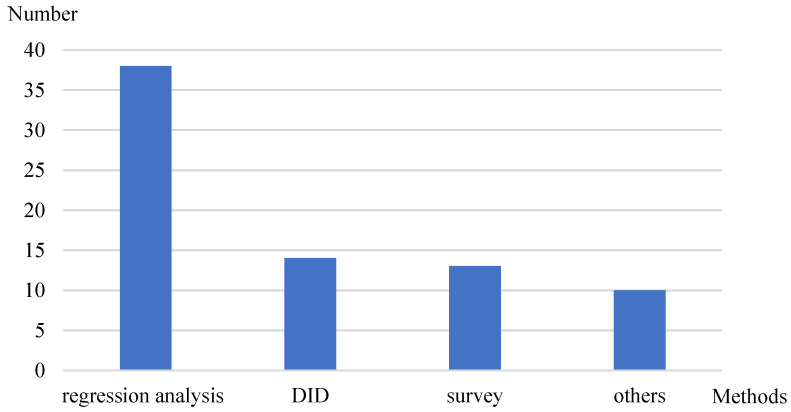
Distribution of research methods.

The included papers employed methods such as regression analysis, difference-in-differences, surveys, and other methods. As shown in Figure 4, the majority of the papers utilized regression analysis, accounting for more than half of the total; 18.67% employed difference-in-differences (DID); 17.33% used survey; and 13.33% applied other methods such as machine learning, experiments, and qualitative analysis.

### 3.3. Themes of the Included Papers

**Figure 5:**
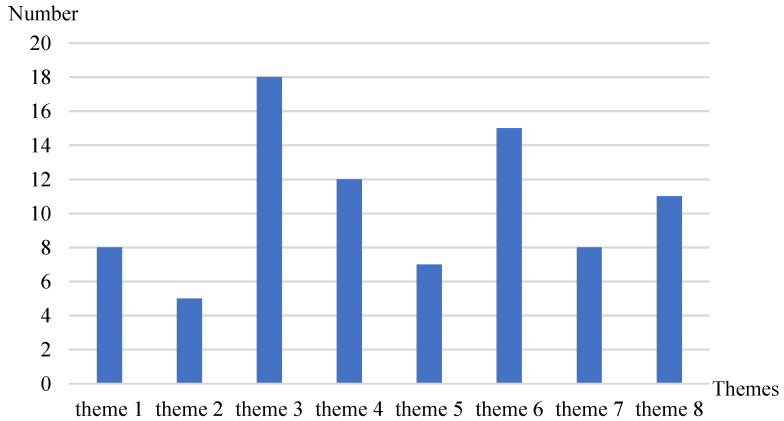
Distribution of research themes.

The distribution of research themes for this systematic review is illustrated in Figure 5. The articles included in the review predominantly focused on the theme of patient choice of doctors (theme 3), with 18 articles addressing this theme. The next most common theme was service quality for patients (theme 6), covered by 14 articles. This indicates that, in online medical consultations, doctor selection and service quality are primary concerns for both patients and researchers. The provision of services by doctors (theme 4) and the spillover effects between online and offline channels (theme 8) were also significant topics, with 12 and 11 articles, respectively. This emphasis is likely due to the foundational role of doctor services in online medical consultations and the transformative impact of online consultations on healthcare practices. Additionally, there are eight articles each on patients utilizing online medical platforms (theme 1) and rewards to doctors (theme 7). There are seven articles on online reviews of patients (theme 5) and five articles on doctors participating in online medical platforms (theme 2).

## 4. Discussion

### 4.1. Online Medical Consultation Starting Point

Online medical consultation functions as a bilateral marketplace involving various participants, and the engagement of these participants serves as the foundational element for the evolution and progress of online medical consultation. This encompasses the utilization of online medical platforms by patients seeking healthcare services, as well as the active involvement of doctors in these digital platforms.

#### 4.1.1. Patients Utilizing Online Medical Platforms

(1) Facilitators

Online medical consultation as a credit service that affects people’s lives and health [17]. Users’ adoption and continued use of online medical consultation are inevitably influenced by their trust in the platform, doctors, and services. The usability and ease of use of online medical consultation websites play a crucial role in establishing user trust [18]. Additionally, social influence indirectly impacts the adoption of online medical consultation by influencing patient trust in healthcare providers [19,20]. Factors such as emotional support and information support [21], timely response rates, the number of thank-you letters received, and electronic word-of-mouth contribute to the perception of service quality, which subsequently affects patients’ perceived usefulness, satisfaction, and willingness to continue using online medical consultation [20,22]. Additionally, doctor engagement and incentive levels for patients in online medical communities also influence patient participation [9,23].

(2) Inhibitors

Despite the extensive research on the facilitators of using online medical consultation, there is a notable lack of investigation into the inhibitors [24]. Inhibitors to the use of online medical consultation can be classified into two primary types. Firstly, individuals may fail to recognize the need to assess the benefits and costs associated with their health behaviors [25]. This phenomenon is evident in offline healthcare habits, where habituated behavior leads individuals to engage in automatic processing without the motivation to change their habits [26]. Considering online medical consultation as a specific form of e-commerce, individuals’ ingrained habit of relying on offline service channels results in a lack of knowledge and awareness regarding the advantages of online healthcare services. Consequently, this lack of awareness negatively impacts their willingness to adopt online service channels [19,24]. The second type of inhibitor arises from individuals perceiving the costs to outweigh the benefits in making rational decisions. For instance, the sunk costs associated with investing in traditional healthcare services, the transition costs involved in shifting from traditional to online medical consultation, and the potential costs associated with privacy protection may outweigh the benefits derived from using online medical consultation [24]. Consequently, these factors hinder users’ willingness to engage with online medical services.

#### 4.1.2. Doctors Participating in Online Medical Platforms

Online medical consultation platforms function as doctor-orientation online health communities, where doctors utilize their available time to address patients’ health inquiries, disseminate medical and health-related knowledge, offer efficient and cost-effective medical services, and assist users in discerning valuable information from misleading sources [27]. Consequently, the quantity and quality of doctors significantly impact the quality of service and the number of patients in online health communities [9]. Therefore, the sustainable growth and development of online health communities rely on the active involvement and continuous contribution of doctors, ensuring ample resources for user communication and consumption [28].

The active participation of doctors in online medical consultations can be challenging and burdensome due to their demanding offline workloads, which may increase the risk of burnout. Therefore, it is crucial to understand the motivations that drive doctor engagement behaviors [29,30]. Motivation theory posits that individuals’ behaviors are influenced by intrinsic and extrinsic motivations [31]. Intrinsic motivations that encourage doctors to contribute to an online doctor–patient community include intellectual self-efficacy, self-value, altruistic motives, empathy, helping motivations, and moral obligations [32,33,34,35]. On the other hand, extrinsic motivations encompass both tangible and intangible factors. The primary extrinsic motivators for doctors to participate in online medical consultations are economic rewards and social rewards. Economic rewards comprise consultation fees, virtual gifts, and bonuses, fulfilling doctors’ financial needs. Social rewards encompass prestige, reputation, word of mouth, and trust [35]. These social rewards are reflected in metrics such as the number of thank-you letters, patient views, likes, and virtual gifts received by doctors [36], satisfying doctors’ social needs as professionals. Social rewards demonstrate professional competence and service quality, which attract more patients, and is particularly important for doctors [37]. For instance, Liu et al. (2020) suggest that incorporating gamification elements into platforms can enhance doctors’ engagement by augmenting their social rewards, and intrinsic motivations such as fun and enjoyment of the gamified experience can also increase engagement. However, it should be noted that gamification design may widen the gap in economic rewards [38].

### 4.2. Online Medical Consultation Process

The patient chooses a doctor online, the doctor provides a service to the patient, the patient reviews the doctor, and the reviews are used as the basis for the patient’s choice of doctor, thus constituting a cyclical process of online medical consultation.

#### 4.2.1. Patient Choice of Doctor

Online medical consultation represents a distinctive service by several key factors. Firstly, it falls under the category of expert services, where the service provider possesses specialized knowledge and information that exceeds that of the service obtainers, resulting in a significant information asymmetry between doctors and patients [9]. Secondly, medical services inherently involve individuals’ lives and well-being, rendering their impact highly consequential. Furthermore, online medical consultation entails an interactive process between service providers and obtainers, making it a credit offering. Consequently, patients must carefully evaluate the service quality and consider the reputation of the doctors when selecting a doctor. To achieve this, patients rely on multiple information sources from various perspectives to assess the service delivery processes and outcomes [39]. There are three primary sources of information that influence patients’ decision-making: doctor-generated information, patient-generated information, and system-generated information [40].

(1) Doctor-generated information

Doctor-generated information refers to the information created through doctors’ online activities and behaviors [40]. Doctors have the ability to update their personal information, share articles, respond to inquiries, and manage patients on their individual websites. This information encompasses various aspects, including personal information, qualities, image, and behavior. Personal information, such as hospital affiliation, specialty, title, city, and working hours, can significantly impact patients’ choices [41]. Personal qualities include the influence of doctors’ competence, benevolence, integrity, and personality similarities between doctors and patients on patient trust and their choice of doctors [11,42,43]. Additionally, the amount of information disclosed by doctors reflects their integrity and can affect patients’ decision-making [42,44]. For personal image, while doctors’ physical attractiveness or beauty do not have a significant impact on patients’ choices, factors like doctors’ smiles and skin conditions positively influence patients’ decisions [45]. Personal behaviors, such as their login patterns, experiences, knowledge shared, prosocial behavior, and level of interaction can also influence patients’ choices [46,47,48,49].

(2) Patient-generated information

Patient-generated information refers to the information created by patients that reflects the quality of a doctor’s services and is closely associated with the doctor’s online reputation [39]. Through sharing their experiences of using and consuming services online, patients contribute to providing others with realistic information about the product or service quality at minimal cost. Patient-generated information encompasses structured numerical ratings and unstructured text reviews [41].

Patient-generated information in the form of structured numerical ratings includes various indicators. These indicators encompass the number of thank-you letters [42,50], virtual gifts [39,42,50], votes [50], service stars [51], online ratings (satisfaction with outcome and satisfaction with attitude) [52], and the total number of patients (number of repeat consultations) [51]. Research has highlighted the significance of these factors as influential signals that affect patients’ decision-making regarding healthcare. Additionally, Yang and Zhang (2019) emphasized that patient-generated paid feedback, such as virtual gifts, had a greater impact on patient choice of doctor compared to free feedback, such as thank-you notes [53].

Unstructured text reviews refer to narrative reviews provided by patients online, offering detailed insights into the quality of a doctor from the patient’s perspective. These reviews serve as vital sources of information for other patients during the decision-making process. Wan et al. (2021) assert that reviews on online medical consultation platforms are highly authentic, specific, and valuable. There are more comprehensive details about the service, diagnosis, and treatment outcomes in the review [54]. The impact of online reviews on patients’ choice of doctor has been examined from various perspectives, including review style, number of reviews [55], review credibility [56], and professional and communicative competence, as well as the technical and functional quality of the service described in the content of the reviews [41,57]. Additionally, doctor popularity has a greater impact on patient choice than service feedback [58].

(3) System-generated information

System-generated information refers to the information produced by a healthcare website system in response to a doctor’s online healthcare service behavior. This information is an estimation generated based on the doctor’s contributions to the website and interactions with patients [39]. The estimation reflects the doctor’s activity level and engagement, indicating their commitment to the online platform. By updating information in a timely manner, publishing articles, educating patients, and responding to consultation questions, a doctor can enhance the value of their contributions [50]. These behaviors serve as indicators of the effort invested by the doctor on the online platform [50] and can provide insights into the quality of their services or behavior [59], assisting patients in selecting the most suitable doctor. While system-generated information does not directly reflect the outcome of a doctor’s service, it does reflect the quality of their service delivery process through the Internet. Moreover, it provides a third-party evaluation that is objective, comprehensive, and accurate [40]. Consequently, it serves as a strong signal for assessing the quality of a doctor’s service [39]. Numerous studies have highlighted the positive influence of system-generated information, such as contribution value and recommendation heat, on patients’ decisions regarding online healthcare [39,40,50,60].

#### 4.2.2. Doctors Providing Services

Doctor participation in online doctor–patient communities involve two primary acts: the voluntary sharing of medical information (referred to as general knowledge sharing) and the fee-based provision of online medical consultation (known as specific knowledge sharing) [61]. General knowledge sharing entails freely sharing health-related educational and professional articles with the public, while specific knowledge sharing involves one-on-one online medical consultations for which a fee is charged [62]. The exchange and sharing of knowledge among community members, including both doctors and patients, play a crucial role in the effectiveness and prosperity of online health communities [63].

As mentioned above, both internal and external motivations serve as significant incentives for doctors to actively engage in online medical consultations and share their knowledge [64]. Yang and Zhang (2019) have observed that economic rewards, such as virtual gifts, exert a greater influence on doctor contributions which include information sharing and consultation services, compared to social rewards, like thank-you notes. Additionally, there is a substitution effect between economic and social rewards [53]. Furthermore, privacy protection and interactions with peer doctors also play a role in doctors’ knowledge sharing. Dang et al. (2020) indicate that privacy protection has a dual impact on professional healthcare knowledge sharing, with a positive effect on interactive professional healthcare knowledge sharing but a negative effect on searching professional healthcare knowledge sharing [65]. Yin et al. (2022) argue that the sharing behavior of peer doctors positively influences the information sharing and consultation service behaviors of focal doctors [61].

Undeniably, there is a higher demand for free services compared to paid services, and these free services play a crucial role in providing users with the necessary information to assess and select doctors [29,30]. Moreover, Meng et al. (2021) have highlighted that the general knowledge-sharing behavior of doctors can impact specific knowledge-sharing behavior through reputation, thereby influencing the growth of online doctor–patient communities [28]. Consequently, incentives for doctors to engage in online free-of-charge service behavior are of particular importance. For instance, Zhou et al. (2019) have observed that both intrinsic motivations (e.g., technical competence) and extrinsic motivations (e.g., online reputation and financial rewards) positively influence the voluntary behavior of mental health service providers. However, the interaction between intrinsic and extrinsic motivations has a negative effect on the voluntary behavior of mental health service providers [30].

#### 4.2.3. Online Reviews of Patients

In the context of online medical consultations, patient behavior can be categorized into consultation and review behavior [60]. Online reviews are often regarded as an authentic representation of patients’ treatment experiences and serve as a written expression of their satisfaction or dissatisfaction with the quality of the doctor’s service. Consequently, online reviews significantly influence potential patients’ perceptions of doctor reputation and their decision-making process [56].

In fact, the provision of online medical services by doctors can directly enhance the review valence provided by patients [66]. For the impact of reviews on doctors, the quantity of reviews is more effective in influencing patient decisions compared to the overall rating of the reviews [67]. The reputation of doctors and their colleagues impacts the number of reviews a doctor receives [68]. When the number of reviews varies, different review styles influence patients’ attitudes toward the doctor differently [55]. Additionally, it is important to note that online reviews are not always completely reliable, and there can be inconsistencies between online reviews and actual service quality, resulting in biases in online doctor reviews [17]. For example, patient perceptions of treatment outcomes are easily influenced by the social impact of experiences shared by other patients [69]. The frequency of interactions, the mode of communication, and the medical information provided can all affect the bias in online doctor reviews [70]. Perceived service quality and uncertainty about service quality can impact the emotional intensity of patient reviews [71].

### 4.3. Online Medical Consultation Outcome

Online medical consultations yield outcomes in the form of patients receiving healthcare services and doctors receiving medical rewards. Moreover, online medical consultations have significant implications for medical ways and healthcare practice.

#### 4.3.1. Service Quality for Patient

Service quality and patient satisfaction have a profound impact not only on patients’ willingness to utilize and continue using online medical consultation platforms, but also on their evaluation and selection of doctors’ services. Consequently, this significantly affects doctors’ earnings and even the overall development of the entire online medical consultation system, which forms the core of the online medical consultation system. Service quality encompasses both the quality of service provided by doctors and the perceived quality of patients, which is reflected in patient satisfaction. The response time and frequency of interaction in the provision of online healthcare services serve as indicators and signals of service quality and have a significant impact on patient satisfaction and doctor–patient relationship [8,72,73].

For the perceived quality of patients, informational support and emotional support are important factors influencing perceived service quality [74], wherein information support exhibits a more pronounced effect compared to emotional support [75]. Specifically, information support exerts a stronger influence on satisfaction with the quality of service, while emotional support has a contrasting effect on satisfaction with the quality of attitude [76]. Patients express their appreciation based on service quality through various means, such as material praise (e.g., virtual gifts) or psychological praise (e.g., thank-you letters) at the different stages of service [77]. Furthermore, due to the inherent information asymmetry between doctors and patients, the evaluation of service quality in online medical consultations often relies on a combination of demonstrative and descriptive signals, with both types of signals exhibiting a complementary and substitute relationship [78].

For the quality of service provided by doctors, professional capital is the foundation of service quality [79]. It is worth noting that the impact of “gift-giving” on the service quality in online medical consultations holds significant importance. The underlying principle of gift theory is the concept of reciprocity, where the recipient of a gift feels a moral obligation to reciprocate. Gift-giving plays a crucial role in facilitating the expression of emotions and building relationships in patient–doctor communication, thereby resulting in higher service quality for patients who engage in gift-giving [80]. Furthermore, the price of the gift has a direct influence on the level of service quality received, with higher-priced gifts correlating to higher levels of service quality [81]. Differentiating between types of gifts, emotional gifts have proven to be more effective in influencing service quality compared to instrumental gifts [80]. Monetary gifts are more effective in encouraging doctors to respond more actively, while digital gifts facilitate follow-up interactions between doctors and patients [82]. Moreover, the act of gift-giving by patients has a spillover effect on the service quality experienced by non-gift-givers. In instances where a patient presents a gift, non-gift-givers may encounter a lower quality of service, characterized by longer waiting times and reduced emotional support [83].

#### 4.3.2. Rewards to Doctors

Ensuring the sustainability of online medical communities requires a balance between benefiting patients and providing incentives for participating doctors [37]. Medical services are characterized as specialized professional services offered by healthcare providers, encompassing both economic transactions and social considerations. The rewards received by participating doctors in these communities can be classified into two primary categories: economic rewards and social rewards. The determinants of doctor rewards are influenced by various factors, including the perspective of doctors themselves, the perspective of patients, and the characteristics associated with the service delivery.

From the perspective of doctors, their professional status and expertise grant them both economic and social benefits within the online medical community [37]. As highly skilled healthcare professionals, doctors possess a unique position and decision-making authority, which can contribute to their overall financial and social returns. Then, considering the patient’s viewpoint, the relationships of patients with doctors can affect economic and social rewards for the doctors [84,85]. Additionally, service quality is a paramount factor influencing the rewards of doctors from the perspective of service-related characteristics [86]. Online medical consultation platforms employ various signals, such as online ratings, thank-you letters, article readings, patient volume, likes, and virtual gifts, to convey service quality to patients [36,60]. Through these signals, doctors’ online reputations are widely acknowledged by patients and society [37]. This recognition not only fulfills doctors’ social reward needs but also positively impacts their economic rewards, resulting in a phenomenon known as the Matthew effect [87]. Furthermore, the mode of service delivery is another factor that influences economic returns. The provision of free services serves as a signal of service quality and thereby generating a positive spillover effect on doctors’ income. However, an excessive number of free services may diminish patient demand for fee-based services [88]. Other service models such as bundling can also positively affect the economic and social returns to doctors [89].

#### 4.3.3. Spillover Effect between Online and Offline Services

The advancement of information technology and the emergence of online medical communities have expanded the channel for patient–doctor interaction, facilitating the coordination and continuity of healthcare. Within the healthcare context, doctors’ online activities can complement their offline professional work, enabling the seamless transition of patients between online consultation and offline treatment, as well as vice versa, thereby promoting continuity of healthcare [90]. Nevertheless, it is important to acknowledge that online and offline channels may not offer identical medical services [67], and considering the limited availability of medical resources (including doctors and their time), there exists a channel effect between online and offline channels that mutually influence each other [91].

Regarding the impact of online channels on offline channels, it is crucial to consider perspectives from both doctors and patients. From the doctors’ standpoint, online channels offer a wealth of medical information, diminish information asymmetry between doctors and patients, and enhance the continuity of information and services. Doctors who actively participate in online medical communities and exhibit greater engagement with online services, such as publishing more articles and responding to patient inquiries, tend to receive more positive electronic word-of-mouth and experience an increase in outpatient visits [2]. This, in turn, leads to higher levels of patient satisfaction, loyalty, and trust [90]. From the patient’s perspective, the quantity of online review comments has a stronger influence on the number of outpatient visits compared to review ratings alone [67], and patient satisfaction with online services also influences their acceptance of offline services [92]. Furthermore, when considering the information provided by doctors on their homepage, patients tend to prefer offline doctors who possess higher-quality subjective information on their homepage rather than relying solely on objective information [93]. Overall, online channels exhibit a complementary effect and enhance doctors’ digital resilience during an epidemic, resulting in a positive spillover effect on offline channels [94,95].

The impact of offline channels on online channels can be examined through the lens of resource-based theory, which posits that individuals must invest various resources, such as time and effort, to reap the benefits of online activities [67]. In the context of healthcare, doctors possess limited time resources, and the allocation of their time and efficiency between online engagement and offline consultations reciprocally influence each other. Constrained by time and resources, an escalation in doctors’ offline outpatient services tends to diminish the volume of online consultation services. However, an increase in the volume of outpatient services prompts doctors to share a greater number of online articles in subsequent periods [88], indicating that their practice-related activities within healthcare institutions accumulate knowledge and materials for their subsequent knowledge sharing on online healthcare platforms. From the patient’s perspective, the patient’s offline experience, satisfaction with offline services, and behavior inertia will influence their access to healthcare services through online channels [96,97].

Online and offline healthcare services exhibit a reciprocal spillover effect and mutually reinforcing interaction. The availability of information regarding both online and offline healthcare services enhance the impact of the quality of healthcare services provided by doctors on patients’ healthcare decisions, thereby positively influencing both patients’ access to offline healthcare and their engagement in online consultations. Furthermore, the integration of online and offline services exerts a positive influence on the demand for and reputation of online healthcare providers [98].

**Figure 6:**
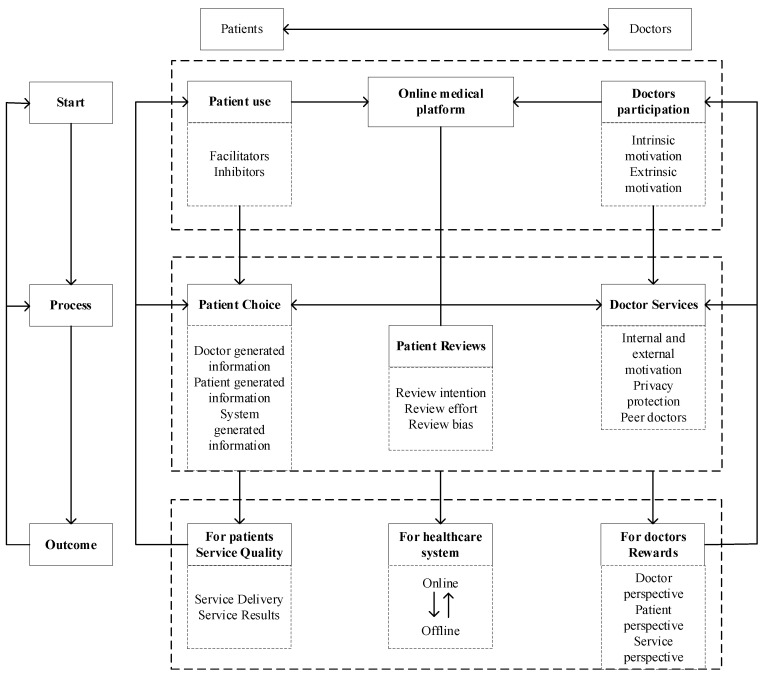
The theoretical framework for online medical consultation research.

The content described above is illustrated in Figure 6, with the relevant literature detailed in the Supplementary Materials.

### 4.4. Future Perspectives

#### 4.4.1. Aspects of Online Medical Consultation Starting Point

Further in-depth exploration is needed to understand the underlying mechanisms that influence patients’ utilization of online medical consultations and doctors’ participation in such consultations.

Existing studies suggest that patient facilitators for using online medical consultations primarily stem from trust in online healthcare, while inhibitors predominantly include habit and cost factors. However, these studies have predominantly focused on the general patient population or specific patient groups, neglecting the nuanced effects of facilitators and inhibitors on the intention to use online medical consultations among patients with distinct characteristics. Future research could delve into the intricate mechanisms of influence between various factors and patients’ willingness to use online medical consultations, taking into account boundary conditions such as patient age, disease type, and health literacy.

In regard to doctor participation, existing research has primarily focused on intrinsic and extrinsic motivations, particularly financial and social rewards, as the main incentives for their engagement in online medical consultations. However, this focus primarily addresses the reward factors for doctor participation while overlooking the contributing factors, the online work environment, and the doctors themselves. This is considering the limitations of the resources they can provide for medical services, particularly when they need to balance online and offline work dynamics. Thus, it is crucial to examine the impact of factors such as personal resources, work resources, and boundaries on doctors’ involvement in online medical consultations.

#### 4.4.2. Aspects of Online Medical Consultation Process

Through an extensive review of existing literature, this study provides a synthesis of the online medical service process, comprising patient choice, doctor service, and patient review. A comprehensive understanding of the interaction process between doctors and patients is vital for optimizing the delivery and outcomes of online medical services. Therefore, it is necessary to continue to explore the process of doctor–patient interaction.

Firstly, the consultation interaction process plays a crucial role in facilitating effective doctor–patient communication. The consultation record serves as a valuable resource for patients to assess the quality of doctors by providing insights into the content and dynamics of the interaction [90]. Therefore, a thorough investigation into the language, emotion, status, and content of the consultation records is warranted to enhance our understanding of this process.

Secondly, the live interaction process, specifically through live-streaming, has emerged as a popular online behavior. Although live-streaming of doctors’ consultations differs from e-commerce live-streaming in terms of generating substantial sales and revenue, it holds significant potential in attracting website traffic, increasing user engagement, shaping doctors’ professional image, and bolstering their online reputation [99]. Consequently, the impact and implications of live-streaming as a medium for doctor–patient interactions require careful examination and consideration.

Thirdly, generative AI, trained on extensive datasets, can generate responses similar to those provided by doctors, thereby assisting doctors in delivering services to patients [100]. However, questions remain regarding whether the emergence of generative AI will attract more patients to use online medical consultations or reduce their use by substituting human doctors. Additionally, it is unclear whether generative AI will enhance or diminish patient satisfaction. Further investigation is needed to understand how generative AI can more effectively provide services to patients.

#### 4.4.3. Aspects of Online Medical Consultation Outcome

The outcomes of online medical consultations warrant in-depth exploration at the micro, meso, and macro levels. At the micro level, there is a need to further investigate the continuity of healthcare between online and offline healthcare and the conflicts that may arise for doctors between their online and offline work [67]. Despite the potential benefits of maintaining continuity of healthcare with traditional brick-and-mortar hospital services, many doctors and hospitals exhibit reluctance in adopting online medical consultations due to the inherent conflicts between online and offline practices. Exploring the impact of online medical consultations on hospital performance and patient health can provide valuable recommendations for hospitals considering the adoption of online medical consultation services [90].

At the meso level, it is essential to examine the role of online medical consultations in optimizing the allocation of healthcare resources on the Internet. This involves investigating whether online medical consultations contribute to supplementing healthcare resources in medically underserved areas or if they pose a threat to the revenues of lower-rated brick-and-mortar hospitals. Additionally, exploring strategies for optimizing the allocation of Internet healthcare resources is a critical research area for the future.

At the macro level, the focus shifts to the role of online medical consultations in the innovative ecosystem of Internet healthcare services. Unlike the traditional healthcare ecosystem, which operates at a relatively slow pace with public brick-and-mortar hospitals at its core, the Internet healthcare service innovation ecosystem enables the coordinated development of various healthcare segments and facilitates progress in the healthcare industry. In this context, online medical consultations serve as the entry point for Internet healthcare traffic and possess the advantages of a network platform. Thus, they play a catalytic role in driving the development of the Internet healthcare service innovation ecosystem.

### 4.5. Practice Proposals

Online medical consultation is a sociotechnical system in which technical systems (such as online medical platforms) and social systems (including doctors and patients) are integrated. The sociotechnical system framework requires that the design and implementation of technical systems align with the needs and characteristics of social systems [101]. Most of the studies included in the systematic review focus on the social aspects of online medical consultations, providing practical proposals for optimizing the technical systems involved.

For patients utilizing online medical platforms, trust and cost are key social factors influencing their use of online medical consultations [18,24]. The platform should provide a user-friendly interface and personalized services to reduce usage difficulty and transition costs, and enhance patient trust through transparent information and high service quality. For doctors participating in online medical platforms, in addition to motivation for participation, factors such as trust in the platform and the doctor’s workload also affect their involvement [88]. Therefore, the platform should include features such as appointment management, medical record access, and remote diagnostic tools, integrate technically with hospital information systems, reduce doctors’ workload, and increase their trust in the technology.

For patient choice of doctor, social factors include information generated by doctors, patients, and the system [40]. Consequently, the platform should feature robust evaluation systems and search and filter functionalities to help patients select suitable doctors based on personal needs, while clearly displaying doctors’ qualifications and platform evaluation standards to facilitate informed decision-making. For doctors providing services, apart from social factors like professional ethics, sufficient technical support, such as medical record management systems and diagnostic aids, can also enhance service quality and efficiency. For online reviews of patients, social factors include patients’ willingness to share reviews and the accuracy of those reviews [17,56]. The platform should optimize the patient review system, strictly vet feedback, and improve both the quantity and quality of reviews.

For the quality of service for patients, which encompasses both the quality of services provided by doctor and patient perceptions of those services, the platform needs to enhance the service delivery system and processes, and use technical means to monitor service quality. For the rewards to doctors, economic and social rewards are significant motivators for providing medical services and are crucial for the sustained development of online medical platforms [84,85]. The platform should design a fair and transparent reward system, incorporating performance tracking tools and rating systems to incentivize high-quality service while avoiding the potential negative effects of reward mechanisms. For the spillover effects between online and offline services, integrating online and offline services helps patients access efficient and cost-effective care [67,90]. Therefore, improving data-sharing systems and patient-referral systems between online and offline services is essential to ensuring continuity and coordination of care.

### 4.6. Limitations

The current synthesis may have some limitations: (1) The exclusion of non-English literature might result in data omission. (2) In order to ensure the quality of the articles included in the systematic review, it is possible that not all studies in the field of online medical consultation were included. (3) This search was conducted on 16 December 2023, and as the literature on online medical consultations continues to grow, further reviews will be necessary. (4) The literature was searched for only in a limited number of databases, which may affect the comprehensiveness of the literature retrieval.

## 5. Conclusions

In order to provide a comprehensive understanding of online medical consultation, we retrieve, screen, and analyze articles related to online medical consultations. According to PRISMA guidelines, a systematic search was conducted in Web of Science, EBSCO, ScienceDirect, PubMed, and Scopus, retrieving a total of 4072 English records. After rigorous screening, 75 articles were included in this review. Then, we outlined the research themes into patients utilizing online medical consultation, doctors participating in online medical consultation, patient choice of doctors, doctors providing services, online reviews of patients, service quality for patients, rewards to doctors, and the spillover effect of online and offline medical services. These themes form the start, process, and outcomes of online medical consultation within the theoretical framework. The start of online medical consultation serves as a necessary precondition for the initiation of the online medical process, which, in turn, generates positive outcomes for patients, doctors, and the platform. Subsequently, these outcomes influence the formation of the start and the development of the process, thus contributing to the continuous advancement of online medical consultation. Lastly, building upon the theoretical framework and previous research, this paper deliberates on the future research directions pertaining to the inception, process, and outcomes of online medical care in the context of online medical consultation.

## Data Availability

No new data were created or analyzed in this study.

## Supplementary Materials

The following supporting information can be downloaded at: https://www.mdpi.com/article/10.3390/healthcare12171687/s1, Table S1: Patients utilizing online medical platforms; Table S2: Doctors participating in online medical platforms; Table S3: Patient choice of doctor; Table S4: Doctors providing services; Table S5: Online reviews of patients; Table S6: Service quality for patients; Table S7: Rewards to doctors; Table S8: Spillover effect between online and offline services.
