# Supplementary material for: A Systematic Review of Online Medical Consultation Research
Source: Healthcare (Basel). 2024 Aug 23;12(17):1687. doi: 10.3390/healthcare12171687 (PMC11394778; doi:10.3390/healthcare12171687)
Supplement: Supplementary file 1 [file healthcare-12-01687-s001.zip › healthcare-3137135-supplementary.pdf]

Table S1. Patients utilizing online medical platforms

Title	Authors	Objective	Method	Journal	Mian finds
“usability + usefulness = trust”: an exploratory study of Australian health web sites	Fisher et al.2008	Explore users’ reactions to health information web sites from the perspective of trust, retrieval of relevant information and ease-of-use	Survey	Internet Research	The website's usability, ease of use, and overall design influence user adoption.
Expert and experiential knowledge in the same place: patients' experiences with online communities connecting patients and health professionals	Vennik et al.2014	Explore patients’ experiences in online health communities	Qualitative content analysis	Patient Education and Counseling	Patients' experiences are that physicians can provide ‘reliable’ and evidence-based information, while patients add experience-based information.
Exploring the inhibitors of online health service use intention: a status quo bias perspective	Zhang et al.2017	Explore the inhibitors of online health service use intention	Survey	Information & Management	Sunk costs, health service habits, transition costs and privacy protection decrease usage intention.
Patient continued use of online health care communities: web mining of patient-doctor communication	Wu 2018	Identify the factors that drive patients to continue their use of online health communities where doctor-patient communication occurs	Structural equation modeling	Journal of Medical Internet Research	Social support, information quality, and service quality positively affect satisfaction and perceived usefulness, which in turn influence continuance use.
Factors influencing the adoption of online health consultation services: the role of subjective	Gong et al.2019	Investigate influencing factors and paths of people’s intention of adopting OHCs.	Survey	Frontiers in Public Health	Subjective norm, trust in providers and perceived benefit positively affect the intention to adopt OHCs, while offline

norm, trust, perceived benefit, and offline habit					habit has a negative effect.
Influencing factors of continuous use of web-based diagnosis and treatment by patients with diabetes: model development and data analysis	Ju et al.2020	Investigate the influencing factors of patients' continuous use of web-based diagnosis and treatment	Regression analysis	Journal of Medical Internet Research	The timely response rate, the reference group, and the number of thank-you letters and patients' ratings are all positively related with the continuous use.
The effects of participating in a physician-driven online health community in managing chronic disease: evidence from two natural experiments	Liu et al.2020	Test the mutual impact between patients' and physicians' participation in OHC.	DID	MIS Quarterly	Physicians' participation significantly increases patients' participation for both diabetes and depression, but patients' participation only increases physicians' participation for depression.
User engagement and user loyalty under different online healthcare community incentives: an experimental study	Shao et al.2022	Examined the impacts of different incentive levels including identity incentive, privilege incentive, and material incentive on user perceived value, user engagement, and user loyalty.	Survey	Frontiers in Psychology	Affective support value and self-health management value demonstrate more importance for user engagement and user loyalty.

Table S2. Doctors participating in online medical platforms

Title	Authors	Objective	Method	Journal	Mian finds
The effects of participating in a physician-driven online	Liu et al.2020	Test the mutual impact between patients' and physicians' participation in OHC.	DID	MIS QUARTERLY	Physicians' participation significantly increases patients'

health community in managing chronic disease: evidence from two natural experiments					participation for both diabetes and depression, but patients' participation only increases physicians' participation for depression.
Understanding the factors influencing health professionals' online voluntary behaviors: evidence from yixinli, a Chinese online health community for mental health	Zhou et al. 2019	Explore how intrinsic and extrinsic motivations influence mental health service providers' voluntary behaviors.	Poisson regression model	International Journal of Medical Informatics	Technical competence, online reputation, and economic rewards positively influence mental health service providers' voluntary behaviors
Deploying gamification to engage physicians in an online health community: an operational paradox	Liu et al. 2020	Examines both positive and negative effects of gamification on physicians and the contingent role of disparity in professional seniority.	DID	International Journal of Production Economics	Although gamification design engages physicians and increases their income, the results indicate increased economic inequality among the physicians as a consequence.
The impact of monetary incentives on physician prosocial behavior in online medical consulting platforms: evidence from China	Jing et al.2019	Explore the effects of monetary incentives on physician prosocial behavior.	DID	Journal of Medical Internet Research	The introduction of monetary incentives had a positive effect on physician prosocial behavior.

How doctors gain social and economic returns in online health-care communities: a professional capital perspective	Guo et al.2017	Examine the determinants of social and economic returns of doctors at OHCs.	Regression analysis	Journal of Management Information Systems	Status capital and decision capital are crucial professional components in sustaining OHC exchange returns.

Table S3. Patient choice of doctor

Title	Authors	Objective	Method	Journal	Mian finds
Investigating the effect of service feedback and physician popularity on physician demand in the virtual healthcare environment	Shah et al.2022	Examines the effects of service feedback and physician popularity on physician demand in virtual healthcare.	DID	Information Technology & People	Physician popularity has a stronger positive effect on demand than service feedback, and this effect is amplified by disease risk.
Achieving popularity to attract more patients via free knowledge sharing in the online health community	Ouyang et al.2023	Examine how physicians' popularity is influenced by their free knowledge sharing, moderated by professional capital.	Regression analysis	Aslib Journal of Information Management	The quantity and quality of articles positively affect physicians' popularity, and the effect is strengthened by physicians' professional capital.
You look like me, and I like you: an empirical study on the influence of doctor-patient personality similarity on patient selection	Chen et al.2023	Explores how doctor and patient personality traits, patient stress, and doctor's title influence patient selection behavior.	Regression analysis	Journal Of Electronic Commerce Research	Doctor-patient personality similarity drives patient selection, with patient stress and doctor's title enhancing this effect.

Factors that determine a patient's willingness to physician selection in online healthcare communities: a trust theory perspective	Gong et al.2021	Explored the influence of physicians' personal quality and online reputation on patients' selection.	Regression analysis	Technology In Society	Physicians' quality (competence, benevolence, integrity) and online reputation (reviews, ratings) significantly affect patient selection.
Patients need emotional support: managing physician disclosure information to attract more patients	Ouyang et al.2021	Investigate the impact of physician self-disclosure on patient decisions in online health communities and the moderating effect of physician online reputation.	Regression analysis	International Journal of Medical Informatics	The emotional orientation in physician self-disclosure positively influences patient decisions; excessive information quantity and topic diversity hinder patient decisions.
Physician's online image and patient's choice in the online health community	Ouyang and wang 2022	Assess the impact of physicians' image on patients' choices in online health communities.	Regression analysis	Internet Research	Physician beauty doesn't significantly affect patient choice; smile and skin status positively affect patient choice.
What influences patients' willingness to choose in online health consultation? An empirical study with PLS-SEM	Wan et al.2020	Explore how physicians' attributes impact patients' trust and their willingness to choose.	Survey	Industrial Management & Data Systems	Physicians' ability positively influenced patients' cognitive trust; integrity and benevolence influenced affective trust. Both types of trust positively impacted patients' choice willingness.
The effect of technical and functional quality on online physician selection:	Fang et al.2022	Test the impact of these service quality signals on online physician selection.	Regression analysis	Information Processing & Management	Technical and functional quality cues are important market signals used by patients to select online physicians.

moderation effect of competition intensity					
Influencing factors and mechanism of doctor consultation volume on online medical consultation platforms based on physician review analysis	Wan et al.2021	Reveal the factors patients consider when choosing a doctor for consultation on an online medical consultation platform.	Regression analysis	Internet Research	Patients mostly care about features such as trust, phraseology, overall service experience, word of mouth and personality traits.
The effect of online effort and reputation of physicians on patients' choice: 3-wave data analysis of China's good doctor website	Deng et al.2019	Examine whether the physicians' online efforts and their reputation have a relationship with patients' choice of physician on PRWs.	Regression analysis	Journal of Medical Internet Research	Physicians' effort positively affected online patient numbers across all models. Measures of physicians' reputations showed varied results.
Converting visitors of physicians' personal websites to customers in online health communities: longitudinal study	Chen et al.2020	Analyze the conversion rate of online health communities and to explore the effects of multisource online health community information.	Regression analysis	Journal of Medical Internet Research	Multisource online health community information positively affected the conversion rate.
Exploring the impact of online information signals in leveraging the economic	Shah et al.2019	Examine the effect of different signals, i.e., patient-generated signals and system generated signals, on patient choice.	Regression analysis	Journal of Biomedical Informatics	Both patient-generated signals and system generated signals have a significant impact on patient choice at

returns of physicians					different stages of health consultation.
Exploring the effects of patient-generated and system-generated information on patients' online search, evaluation and decision	Yang et al.2015	Verify the effects of two kinds of information on patients' search, evaluation and decision-making on healthcare websites.	Regression analysis	Electronic Commerce Research and Applications	Positive patient-generated and system-generated information on physicians' service quality positively influences patient reactions across various stages.
Insights into the impact of online physician reviews on patients' decision making: randomized experiment	Grabner-kräuter and Waiguny 2015	Explore how certain characteristics of physician reviews affect the evaluation of the review and users' attitudes toward the rated physician.	Experiments	Journal of Medical Internet Research	Link the review style (factual vs. Emotional) and review quantity (many vs. Few) to the structure of review acceptance.
Investigating the effect of paid and free feedback about physicians' telemedicine services on patients' and physicians' behaviors: panel data analysis	Yang and Zhang 2019	Investigate the impact of free and paid feedback on patient choices and physician behaviors, and explore their substitution relationship in telemedicine markets.	Regression analysis	Journal of Medical Internet Research	Paid feedback significantly influences patient choice and physician contribution in telemedicine markets more than free feedback.
Impact of a physician's prosocial behavior on the patient's choice: an empirical	Wang et al.2023	Explore the influence of a physician's prosocial behavior on a patient's choices in the online health community context.	DID	Information Technology & People	A physician's prosocial behavior improves a patient's choice only when the strength of a physician's prosocial

investigation in online health community					behavior is below the tipping point
Better interaction performance attracts more chronic patients? Evidence from an online health platform	Liu et al.2023	Investigates how a physician's OHC performance influences patients' choice of consulting the physician in a chronic disease context on an online health platform.	Regression analysis	Information Processing & Management	Better performance in the interaction breadth and length helps physicians attract more patients while higher interaction depth reduces patients' choice of physician.
Online selection of a physician by patients: empirical study from elaboration likelihood perspective	Cao et al.2017	Considered service quality as the central route, and electronic word-of-mouth (eWoM) as the peripheral cue, and explored their importance.	Regression analysis	Computers in Human Behavior	Service quality and eWoM both had positive effects on patients' selection decisions. Disease knowledge increased the importance of service quality on patients' choices.

Table S4. Doctors providing services

Title	Authors	Objective	Method	Journal	Mian finds
The effects of participating in a physician-driven online health community in managing chronic disease: evidence from two natural experiments	Liu et al.2020	Test the mutual impact between patients' and physicians' participation in OHC.	DID	MIS Quarterly	Physicians' participation significantly increases patients' participation for both diabetes and depression, but patients' participation only increases physicians' participation for depression.
Investigating the effect of paid and free feedback about physicians' telemedicine	Yang and Zhang 2019	Investigate the impact of free and paid feedback on patient choices and physician behaviors.	Regression analysis	Journal of Medical Internet Research	Paid feedback significantly influences patient choice and physician contribution in

services on patients' and physicians' behaviors: panel data analysis					telemedicine markets more than free feedback.
Converting readers to patients? From free to paid knowledge-sharing in online health communities	Meng et al.2021	Explore the relationship between general and specific knowledge-sharing.	Regression analysis	Information Processing and Management	General knowledge-sharing is positively associated with specific knowledge-sharing, and this effect is exerted through online reputation.
Social capital, motivations, and knowledge sharing intention in health Q&A communities	Zhang et al.2017	Examine how social capital and motivation influence knowledge sharing in health Q&A communities among health professionals and users.	Survey	Management Decision	Social capital positively affects intrinsic and extrinsic motivations, which then positively influence the intention of health professionals and normal users to share knowledge.
Knowledge sharing motivations in online health communities: a comparative study of health professionals and normal users	Zhang et al.2017	Examines both the extrinsic and intrinsic motivations of health professionals and normal users.	Survey	Computers in Human Behavior	Results indicate that reciprocity and altruism positively impact knowledge sharing intentions among health professionals and normal users.
The impact of monetary incentives on physician prosocial behavior in online medical consulting platforms: evidence from China	Jing et al.2019	Explore the effects of monetary incentives on physician prosocial behavior and investigate the moderation effects of self-recognition and recognition from others of physician competence.	DID	Journal of Medical Internet Research	The introduction of monetary incentives had a positive effect on physician prosocial behavior. Higher self-recognition and others' recognition level of physician competence increased this promotion effect.

Exploring the peer effect of physicians' and patients' participation behavior: evidence from online health communities	Yin et al.2022	Examines the influence of a focal physician's peers (f-peers) on the physician and the impact of patients of those peers (f-p-patients) on the physician's own patients (f-patients).	Regression analysis	International Journal of Environmental	The behavior of f-peers positively affects the behavior of the f-physician, while the behavior of f-p-patients positively affects the behavior of f-patients.
Privacy protection in online health communities: natural experimental empirical study	Dang et al.2022	Examined the relationship between privacy protection and professional health care knowledge sharing in OHCs.	DID	Journal of Medical Internet Research	Privacy settings significantly enhance interactive professional healthcare knowledge sharing but hinder searching for such knowledge.
Exploring doctors' willingness to provide online counseling services: the roles of motivations and costs	Chen et al.2020	Examine the relationships between anticipated outcomes, performance expectations, and effort intentions from online and offline perspectives.	Survey	International Journal of Environmental Research and Public Health	Extrinsic motivations and intrinsic motivation significantly influence the desire to serve patients well, which in turn positively affects the willingness to offer free services and the willingness to offer paid services.
Understanding the satisfaction and continuance intention of knowledge contribution by health professionals in online health communities	Imlawia and Gregg 2020	Identify the factors that might affect health professionals' intentions to continue contributing health knowledge.	Survey	Informatics for Health & Social Care	Helping motivator, self-efficacy motivator, and reputation motivator affect OHC members' satisfaction.
What motivates physicians to share free health information on	Zhang et al.2020	Explore the influences of material and professional motivation on free	Regression analysis	Information Processing & Management	In addition to material motivation, professional motivation also plays a primary role in

online health platforms		information sharing and the contingent roles of professional expertise and online expertise.			inducing physicians to share free information.
Not just for the money? An examination of the motives behind physicians' sharing of paid health information	Yang et al.2023	Examine how extrinsic motivation, enjoyment, and professional motivation influence the sharing of paid information.	Survey	Journal of Information Science	Extrinsic motivation, enjoyment, and professional motivation significantly influence physicians' inclination to share paid information. Income ratio further moderates these motivational effects on paid information sharing.

Table S5. Online reviews of patients

**Title**	**Authors**	**Objective**	**Method**	**Journal**	**Mian finds**
How your colleagues' reputation impacts your patients' odds of posting experiences: Evidence from an online health community	Wu and Lu 2016	Investigating how the reputation of one physician's colleagues affects the focal physician's future review amount.	Regression analysis	Electronic Commerce Research and Applications	Both the focal physician's reputation and his/her colleagues' reputation have significant impact on his/her patients' odds of sharing their treatment experience online.
Shared Minds: How Patients Use Collaborative Information Sharing via Social Media Platforms	Yan et al.2019	Incorporate treatment uncertainty, information exposure, and information source credibility into patients' information evaluation to assess their use of shared health information and experiences.	Regression analysis	Production and Operations Management	Social influence created by generalized others in the community outweighs that by familiar others of one's intimate social group.

The effect of interactive factors on online health consultation review deviation: An empirical investigation	He et al.2022	Test the effects of interactive factors on online doctor review deviation and to further explore deviation across doctor specialties.	Logistic regression	International Journal of Medical Informatics	Interaction frequency, messaging methods, and medical information influence bias in online doctor reviews.
Understanding online review behaviors of patients in online health communities: an expectation-disconfirmation perspective	Chen et al.2021	Determine the factors influencing patients' online review behavior in OHCs, including the emotional response and reviewing effort.	Regression analysis	Information Technology & People	Service quality perception and disconfirmation positively affect patients' positive emotional intensity in textual reviews, with disease severity strengthening these relationships.
How online reviews and services affect physician outpatient visits: content analysis of evidence from two online health care communities	Lu and Wu 2019	Examined the impact of online reviews and services on outpatient visits.	DID	JMIR Medical Informatics	Compared to overall review ratings, the number of reviews is more effective in influencing patient decisions. Online services moderate the relationship between online reviews and doctors' outpatient visits.
Insights into the impact of online physician reviews on patients' decision making: randomized experiment	Grabner-Kräuter and Waiguny 2015	Explore how certain characteristics of physician reviews affect the evaluation of the review and users' attitudes toward the rated physician.	Experiment	Journal of Medical Internet Research	Link the review style (factual vs. Emotional) and review quantity (many vs. Few) to the structure of review acceptance.
Doctors' Provision of Online Health Consultation Service and Patient Review Valence: Evidence from a Quasi-Experiment	Liu et al.2022	The effects of doctors' provision of online health consultation service (OHCSV) on the review valence provided by patients.	DID	Information & Management	The OHCSV provided by physicians increases the value of patient reviews and significantly alters the value structure by increasing the proportion of positive

					value and decreasing the proportion of negative value.

Table S6. Service quality for patients

Title	Authors	Objective	Method	Journal	Mian finds
Exploring the influence of the online physician service delivery process on patient satisfaction	Yang et al.2015	Investigate the impact of the online doctor's service delivery process on patient satisfaction.	Regression analysis	Decision Support Systems	The online doctor's service delivery process significantly affects patient satisfaction, particularly in terms of response speed and interaction frequency.
Consequences of gift giving in online health communities on physician service quality: empirical text mining study	Peng et al.2020	Addressed the consequences of gift giving by mining and analyzing the dynamic physician-patient interaction processes.	Regression analysis	Journal of Medical Internet Research	Patient gift giving inspires physicians to improve their service quality, affective gifts and instrumental gifts had different effects in improving physicians' service quality online.
Quid pro quo in online medical consultation? Investigating the effects of small monetary gifts from patients	Zhao et al.2022	Investigates whether small gifts from patients impact the quality of service provided to the gift-givers and the nongivers.	Regression analysis	Production and Operations Management	Doctors who receive gifts do reciprocate to the gift-givers by providing them with more timely responses and greater emotional support.
Online service qualities in the multistage process and patients' compliments: a transaction cycle perspective	Wu et al.2020	Explore how a patient's choice for psychological and material compliments is affected by service quality received at different stages.	Regression analysis	Information & Management	High service quality at the pre- and after-sale stages motivated patients to give material compliments rather than psychological compliments, whereas psychological compliment is more likely to be triggered for high service

					quality received at the during-sale stage.
A hybrid IT framework for identifying high-quality physicians using big data analytics	Ye et al.2019	To address patients' information needs holistically, we propose a four-dimensional IT framework based on signaling theory.	Machine learning	International Journal of Information Management	The model takes expertise knowledge, online reviews, profile descriptions and service quality as signals that distinguish high-quality physicians.
Exploring the online doctor-patient interaction on patient satisfaction based on text mining and empirical analysis	Chen et al.2020	Explore the influences of online doctor-patient interaction content on patient satisfaction.	Regression analysis	Information Processing & Management	Patient engagement positively influences both informational and emotional support provided by doctors, which positively affect patient satisfaction.
Impact of the price of gifts from patients on physicians' service quality in online consultations: empirical study based on social exchange theory	Wang et al.2020	Explore the impact of gift price on the quality of physicians' online consultation service.	Regression analysis	Journal of Medical Internet Research	Gift price had a positive impact on the quality of physicians' online consultation service. This impact was negatively mediated by both the physician's service price and the total number of gifts they received.
Description and demonstration signals as complements and substitutes in an online market for mental health care	Zhou et al. 2022	Determines whether the demonstration signal of responsiveness to client questions substitutes or complements the two description signals of professional qualifications and counseling style.	Zero-inflated negative binomial models	MIS Quarterly	Relative credibility of signals is the boundary condition that determines when a demonstration signal will complement and when it will substitute for a description signal.

Informal payments and doctor engagement in online health communities: an empirical investigation using generalized synthetic control	Wang et al.2023	Examines how informal payments in the form of monetary gifts affect doctor engagement.	DID	Information Systems Research	Monetary and nonmonetary gifts play distinct roles in motivating doctor responses, with nonmonetary gifts having a more significant carryover effect on follow-up interactions and better promoting the doctor-patient relationship.
How does online interactional unfairness matter for patient-doctor relationship quality in online health consultation? The contingencies of professional seniority and disease severity	Zhang et al.2019	examines how online interactional unfairness influences online patient–doctor relationship quality.	Regression analysis	European Journal of Information Systems	Online interpersonal unfairness and online informational unfairness have negative and positive effects on Relationship-quality incentive, respectively.
The impact of individual and team professional capital on physicians' performance in online health-care communities: a cross-level investigation	Wang et al.2023	hypothesize multilevel and cross-level professional capital determinants of physicians' performance.	Regression analysis	Internet Research	Physicians' status capital (SC) and decisional capital (DC) are positively related to their performance.
Does voice matter? Investigating patient satisfaction on mobile health consultation	Liu et al.2023	Examine the relative effects of informational support and emotional support on patient satisfaction and the moderating role of consultation channels	Experiments	Information Processing & Management	Informational support had a more substantial effect on medical quality satisfaction than emotional support, while the impact of the former on service attitude satisfaction was

					weaker than that of the latter.
Satisfaction or gratitude? Exploring the disparate effects of physicians' knowledge sharing on patients' service evaluation in online medical consultations	Tan et al.2023	Distinguish patient satisfaction from gratitude in OMCs and elucidate the relationship between the sharing process and outcomes.	Regression analysis	Information Systems Journal	Patient gratitude is associated with a more favorable service evaluation than satisfaction in OMCs
Physician-user interaction and users' perceived service quality: evidence from Chinese mobile healthcare consultation	Tan and Yan 2020	This study proposes a theoretical model predicting users' PSQ in mobile consultation based on common interactive factors, unique mobile factors, and their interactions.	Regression analysis	Information Technology & People	Physicians' informational, emotional support, response speed and voice service are found to have significant positive impacts on users' PSQ.

Table S7. Rewards to doctors

Title	Authors	Objective	Method	Journal	Mian finds
Do the rich grow richer? An empirical analysis of the Matthew effect in an online healthcare community	Zhou et al.2022	Investigates whether doctors with higher ratings earn more platform income compared with those with lower ratings.	Regression analysis	Electronic Commerce Research and Applications	Online doctor ratings have a significantly positive relationship with doctors' online income composed of consultation fees and patient remuneration.
Impact of Free Contributions on Private Benefits in Online Healthcare Communities	Zhang et al.2019	Investigate the relationship between online free services and the private benefits of online paid services.	Regression analysis	International Journal of Electronic Commerce	Participation in online free services significantly boosts family doctors' private benefits up to a certain point, with doctor ranking moderating this effect.

Analysis of Massive Online Medical Consultation Service Data to Understand Physicians' Economic Return: Observational Data Mining Study	Jiang et al.2020	Identify features linked to patient payment versus free trials, assess their importance, and understand their interactions with payment.	Machine learning	JMIR Medical Informatics	Compared to characteristics associated with doctors' reputation, features related to service appear to contribute more significantly to patients' payment decisions.
How Doctors Gain Social and Economic Returns in Online Health-Care Communities: A Professional Capital Perspective	Guo et al.2017	Examine the determinants of social and economic returns of doctors at OHCs by extending the social exchange theory into the professional domain.	Regression analysis	Journal Of Management Information Systems	Status capital and decision capital are crucial professional components in sustaining OHC exchange returns.
Doctor-patient relationship strength's impact in an online healthcare community	Guo et al.2018	Examines the strength of the D–P relationship and its impacts on physicians' individual outcomes.	structural equation modeling (SEM)	Information Technology for Development	Weak ties can result in economic and social returns for doctors. However, further analysis has indicated that strong ties mediate the effect of weak ties.
Exploring the impact of online information signals in leveraging the economic returns of physicians	Shah et al.2019	Examine the effect of different signals, i.e., patient-generated signals (PGSs) and system generated signals (SGSs), on patient choice.	Regression analysis	Journal of Biomedical Informatics	Both PGSs and SGSs have a significant impact on patient choice at different stages of health consultation. Furthermore, disease risk negatively moderates the association between PGSs and information search.
Effect of New Bundled Services on Doctors' Online Social and Economic Returns: Evidence from an	Yin et al.2023	Investigates the effects of bundling strategy on doctors' online returns in OHCS.	DID	Information Systems Frontiers	The launch of bundled services can positively affect doctors' online reputation and part of separate components. A doctor's tenure also moderates the effects

Online Health Community					of bundled services on doctors' returns.
The effects of social media use and consumer engagement on physician online return: evidence from Weibo	Yang et al.2023	Investigates physicians' online economic and social capital return in relation to physicians' use of social media and consumer engagement.	Regression analysis	Internet Research	Physicians' social media use and consumer sharing enhance their online economic return, while consumer engagement boosts their online social capital return.

Table S8. Spillover effect between online and offline services

Title	Authors	Objective	Method	Journal	Mian finds
How online health community participation affects physicians' performance in hospitals: empirical evidence from China	Wu et al.2021	Examine the digital channel's effect on healthcare by focusing on key indicators of performance: outpatient visits, operational efficiency, resource utilization efficiency, patient loyalty, and satisfaction	DID	Information & Management	A physician who provides online health services has a higher number of outpatient visits, less waiting time, higher patient satisfaction, and loyalty in the offline channel.
Online written consultation, telephone consultation and offline appointment: an examination of the channel effect in online health communities	Wu and Lu 2017	Examines how online channel usage affects offline channels, and how the channel effects change with doctors' online and offline reputation.	Regression analysis	International Journal of Medical Informatics	Written consultations supplement OBSH, while telephone consultations substitute OBSH. Doctors with high online and offline reputations can attract more patients to use OBSH.
Exploring the role of online health community information in	Li et al.2019	Investigate how subjective and objective information influence patients'	Regression analysis	International Journal of Medical Informatics	Patients are more likely to switch to offline doctors whose subjective information quality on the

patients' decisions to switch from online to offline medical services		decisions to switch from online to offline medical services provided by doctors.			homepage is higher than the objective information quality.
How online reviews and services affect physician outpatient visits: content analysis of evidence from two online health care communities	Lu and Wu 2019	Examined the impact of online reviews and services on outpatient visits.	DID	JMIR Medical Informatics	Compared to overall review ratings, the number of reviews is more effective in influencing patient decisions. Online services moderate the relationship between online reviews and doctors' outpatient visits.
Understanding physicians' online-offline behavior dynamics: an empirical study	Wang et al.2020	Investigate physicians' online-offline behavior dynamics using data from both online and offline channels.	Structural vector autoregression (SVAR)	Information Systems Research	Doctors' online activities can increase the volume of services through offline channels, while offline activities may reduce doctors' online services due to resource constraints.
How does online doctor-patient interaction affect online consultation and offline medical treatment?	Xing et al.2019	Investigate factors that influence the patients' intentions to visit doctors face-to-face for consultations.	Survey	Industrial Management & Data Systems	Perceived fairness and service quality positively impact patient satisfaction, while satisfaction and intention to use online consultations positively influence the intention to seek in-person consultations.
Offline to online: the impacts of offline visit experience on online behaviors and service in an internet hospital	Ye and Wu 2023	Investigating the impact of patients' offline visit experience on their online engagement, and then how patients' online engagement affects	Regression analysis	Electronic Markets	Patients' online information provision positively influences physicians' online service quality, while negatively influencing online service efficiency

		the quality and efficiency of physicians' online services			
Should doctors open online consultation services? An empirical investigation of their impact on offline appointments	Fan et al.2022	Examining the overall impact of offering online consultations on offline appointments.	Regression analysis	Information Systems Research	The number of offline appointments for doctors increases after opening an online consultation service.
Understanding the digital resilience of physicians during the covid-19 pandemic: an empirical study	Lin et al.2023	Focuses on the digital resilience of certified physicians who adopted an online healthcare community (OHC) to acquire patients and conduct telemedicine services during the pandemic.	DID	MIS Quarterly	These physicians had 35.0% less reduction in medical consultations in the immediate period and 31.0% more bounce-back in the subsequent period as compared to physicians who did not adopt the OHC.
Effects of online–offline service integration on e-healthcare providers: a quasi-natural experiment	Huang et al.2021	Quantify the effects of online–offline service integration on the e-healthcare providers' demand and reputational outcomes.	DID	Production and Operations Management	E-healthcare providers, on average, experience increases in online demand and decreases in offline demand post online–offline service integration.
Switching from offline to online health consultation in the post-pandemic era: the role of perceived pandemic risk	Pan et al.2023	Measures the users' perceptions towards both offline and online services, their intention to switch to OHC, and the perceived pandemic risks.	Survey	Frontiers In Public Health	Dissatisfaction with offline services, the attractiveness of online health communities (OHCs), and user behavior inertia jointly influence the intention to switch to OHCs.
